# A Case of Advanced Oral Submucous Fibrosis Management: Beyond Conventional Approach

**DOI:** 10.7759/cureus.61916

**Published:** 2024-06-07

**Authors:** Palak Agrawal, Chetan Gupta, Nitin Bhola, Shreya S Pawar, Parmarth M Sonpal, Vaishnavi Gattani

**Affiliations:** 1 Oral and Maxillofacial Surgery, Sharad Pawar Dental College and Hospital, Wardha, IND

**Keywords:** surgical intervention, betel nut, coronoidectomy, fibrotomy, oral submucous fibrosis

## Abstract

A chronic, persistent, possibly cancerous condition that mostly affects the oral cavity is called oral submucous fibrosis (OSMF) and causes severe functional impairment. Due to its complex nature, OSMF requires a comprehensive strategy that includes both surgical and medication therapies. Multidisciplinary treatment was started, which included a complete stoppage of habit, dental hygiene precautions, dietary counselling, surgical intervention, supportive medicinal therapy, and physiotherapy. Following surgery and adjunct therapy, the patient's mouth opening and functional results were improved. The patient is kept for regular follow-up to assess the recurrence of fibrosis or any incidence of malignant transformation. This case emphasizes the difficulties in treating advanced OSMF and emphasizes how crucial it is to improve patient outcomes by early detection, stopping betel nut chewing, and thorough multidisciplinary care.

## Introduction

Oral submucous fibrosis (OSMF) has been traditionally described as “An insidious, chronic mucosal condition affecting any part of oral cavity and sometimes pharynx, associated with fibroelastic transformation of juxtaepithelial connective tissue layer and epithelial atrophy leading to progressive stiffness of the oral mucosa causing trismus and inability to eat” by Joshi et al. in 1950 [1]. The oral mucosa becomes fibrotic and rigid as it mostly affects the submucosal tissues of the oropharynx and oral cavity [2]. In nations like India, Bangladesh, Sri Lanka, and portions of Southeast Asia, oral submucous fibrosis is seen as a serious public health problem, even if exact incidence rates may be difficult to ascertain because of underreporting and variations in diagnostic criteria. It is acknowledged that oral submucous fibrosis is a common and potentially crippling illness in areas where betel quid intake is well embedded in customs and social norms. Different populations have varied incidence rates of oral submucous fibrosis, according to several research. For instance, the reported prevalence of oral submucous fibrosis among adults in areas of India according to 2024 reports where chewing betel quid is common ranges from 0.62% to over 6.42%, with greater rates noted in locations with more betel quid usage [3].

Oral submucous fibrosis is characterized by an accumulation of collagen fibres that leads to a progressive loss of mouth opening, mucosal stiffness, and surface changes which lead to burning sensation and functional impairment which further affects the patient’s quality of life greatly [4]. The precise pathophysiology of oral submucous fibrosis is yet unknown, although it involves immunologic components, genetic predispositions, and the presence of certain carcinogens found in tobacco and areca nut [5]. When it comes to clinical manifestations, OSMF can cause anything from pain to significant limitations in oral function. Patients may first experience oral mucosal burning and ulceration to average spicy food, which can then proceed to limited mouth opening, dysphagia, and trouble speaking [6,7]. Treating oral submucous fibrosis is generally done to reduce the underlying etiological causes, manage symptoms such as burning sensation, trismus, mucosal changes that took place and to prevent the malignant transformation of the condition, and maintain oral function, reduce social discomfort, and quality of life for those who are impacted.

In recent years, significant advancements in the diagnosis and clinical treatment relevant to oral submucous fibrosis have been reported. Treatment options include stopping the usage of betel quid, taking anti-inflammatory and anti-fibrotic drugs, engaging in physiotherapy to increase mouth opening, and in extreme and very advance situations, undergoing surgery to relieve fibrous bands. Effective management of oral submucous fibrosis requires routine monitoring and follow-up [8]. Here, we report the case of a 45-year-old male patient with significant trismus and difficulty in mastication, with advanced oral submucous fibrosis which is treated surgically by a versatile bi-winged nasolabial flap which offers a sustainable, relapse-free, and cost-effective solution for long-term management.

## Case presentation

**Figure 1:**
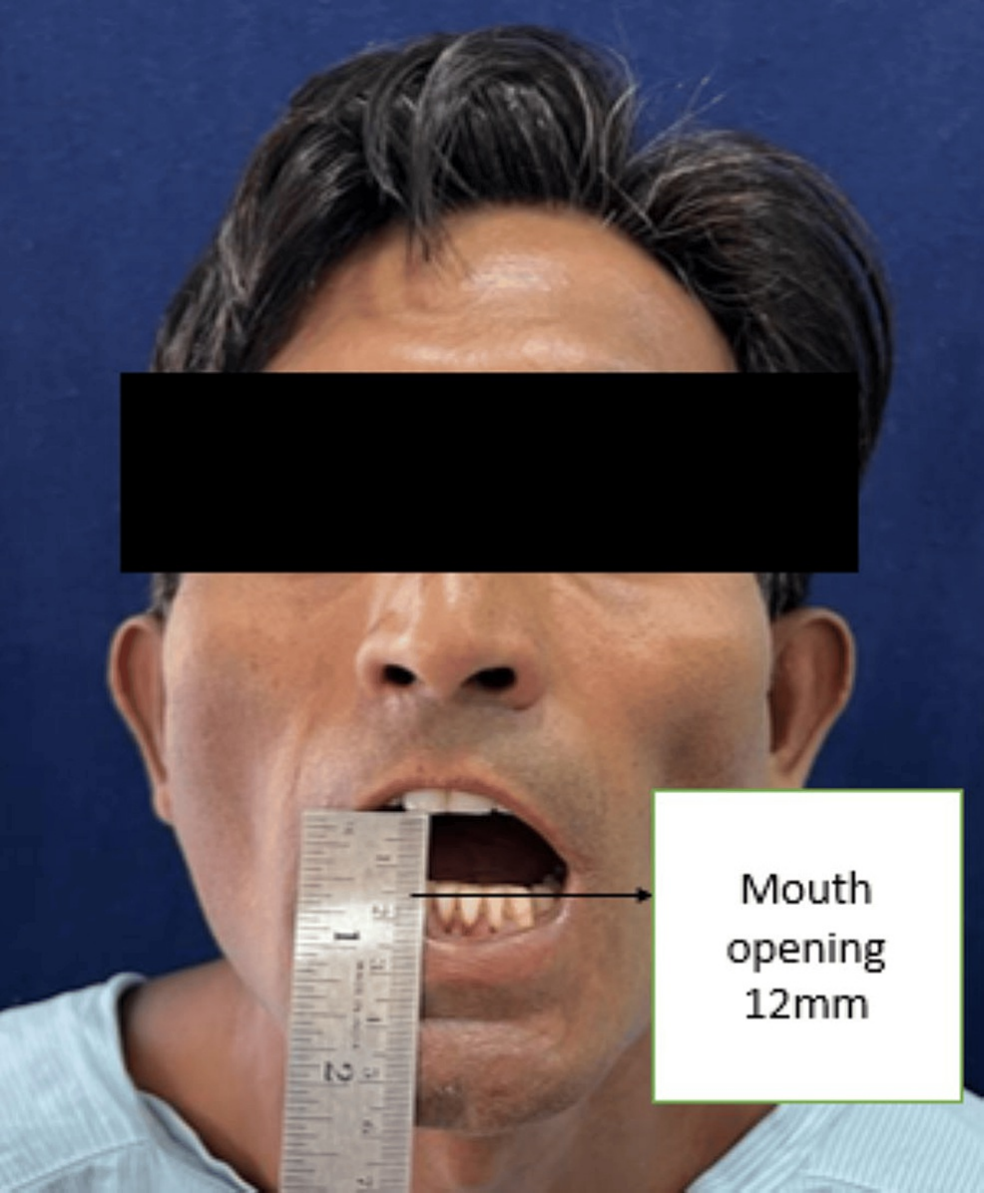
Mouth opening of the patient pre-operatively

**Figure 2:**
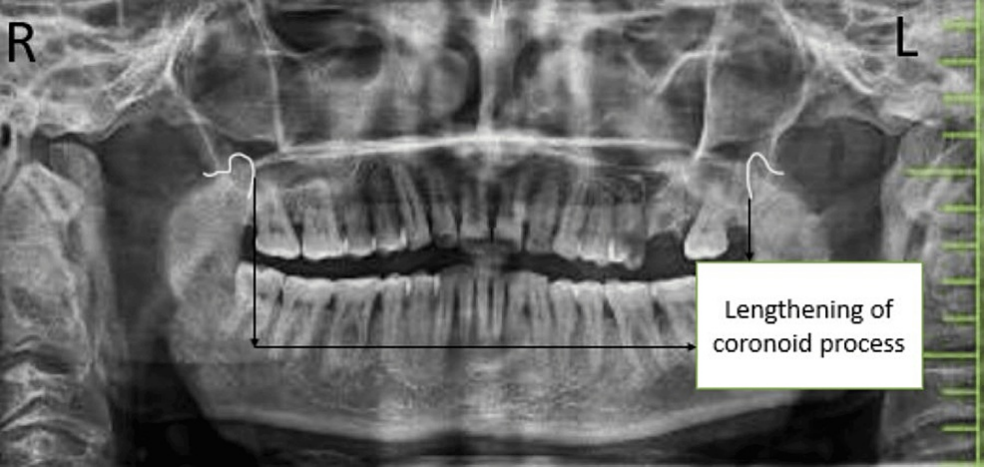
Lengthening of coronoid process due to temporalis muscle pull

A 45-year-old gentleman reported to our outpatient department of Oral and Maxillofacial Surgery in Sharad Pawar Dental College and Hospital with the chief complaint of reduced mouth opening and burning sensation in the oral cavity, which has been gradually progressing for the last four years. During the initial days, the mouth opening was up to three fingers wide, which has gradually reduced to approximately one finger. The patient also experienced a burning sensation after consuming average hot and spicy food for approximately five years. The patient also noticed a change in the consistency of saliva from thin to thick and ropy, which is also associated with difficulty in mastication due to a burning sensation, loss of appetite, and weight loss of around 2-3 kg. The patient also gave a history of difficulty in cheek blowing in the last six months, approximately. A history of bilateral earaches for three months is also present. The patient gives a history of khara chewing eight to 10 times a day for approximately 10 years. The patient had no significant medical history. The examination was initiated after obtaining informed consent. All the vitals of the patient were examined which were in the normal limits. The patient was conscious, cooperative, and well-oriented to time, place, and person during the general examination. The systemic examination was normal. The extraoral examination reveals shrunken cheeks on both sides with reduced cheek blowing but no gross facial asymmetry. Lips are competent. Reduced mouth opening occurs without any limitation in jaw movements. There was reduced mouth opening of about 12 mm (Figure 1). The blanching was present on palate, faucial pillars, retromolar trigone area, and bilateral buccal mucosa. The bud-shaped shrunken uvula present. On palpation, bilateral vertical fibrous bands that include the faucial pillars and extend to the retromolar trigone area are palpable. All routine blood investigations were done, and reports show values in the normal range; radiographic investigations such as orthopantomography and all the necessary clinical findings were noted and shown in Table 1. By clinical and radiographic findings, we got a final diagnosis of grade 4 OSMF. Hard, white fibrous bands were palpated on both the buccal mucosa. The orthopantomograph (OPG) shows lengthening of coronoid process due to the constant temporalis muscle pull (Figure 2).

**Table 1:** All the laboratory values with their normal reference values

Sr no	Investigations	Normal values	Patient’s values
1	Haemoglobin count	14%-18%	13
2	Total red blood cell count	3.7-6.1 cells/μl	3.42 cells/μl
3	Total white blood cell count	5,000-10,000 cells/μl	5,200 cells/μl
4	Haematocrit value	36%-48%	36.6%
5	Mean corpuscular haemoglobin concentration	32-36 g/dl	35.5 g/dl
6	Mean corpuscular volume	80-100 fl	106.9 fl
7	Mean corpuscular haemoglobin	27-31 pg	37.9 pg
8	Total platelet count	1.35-3.17	2.36
9	Red blood cell distribution width	11.5%-14.7%	14.7%
14	Prothrombin time	10-13 seconds	12 seconds
15	Activated partial thromboplastin time	30-40 seconds	30
18	Potassium ions level	3.5-5 mmol	4.7 mmol
19	Sodium ions level	135-145 mEq/l	139
20	Random blood sugar level	82-115 mg/l	89

**Figure 3:**
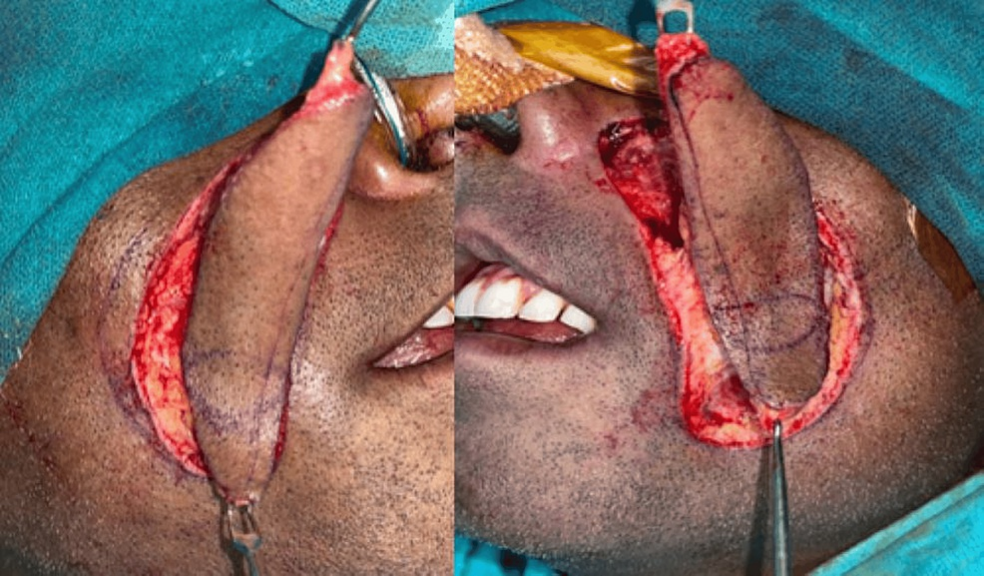
Extraoral markings of the modified nasolabial flap (seagull flap) which was taken for the repair of intraoral defect

After a complete diagnostic evaluation and thorough pre-anaesthetic checkup, the patient was planned for surgical intervention. After the fibreoptic nasoendotracheal intubation, general anaesthesia was induced. The patient was prepared, and an intraoral incision was made 1 cm posterior to the corner of the mouth, extending towards the retromolar trigone, ensuring placement below 5-7 mm to the parotid duct. Bilateral fibrous band was released accompanied by third molar extractions across all quadrants. The inter-incisal mouth opening (IIMO) of 25 mm was achieved. To further improve mouth opening and treat coronoid hyperplasia, bilateral coronoidectomy was performed, achieved an intraoperative IIMO of 40 mm, and the passive mouth opening was about 30 mm. The surgically created gap was resurfaced with bilateral bi-winged nasolabial flap and sutured with resorbable vicryl suture, to maximize functional results and to restore mucosal integrity of skin (Figure 3 shows the kind of flap taken). The extraoral defect was closed primarily in two layered fashions.

Post-operative care

**Figure 4:**
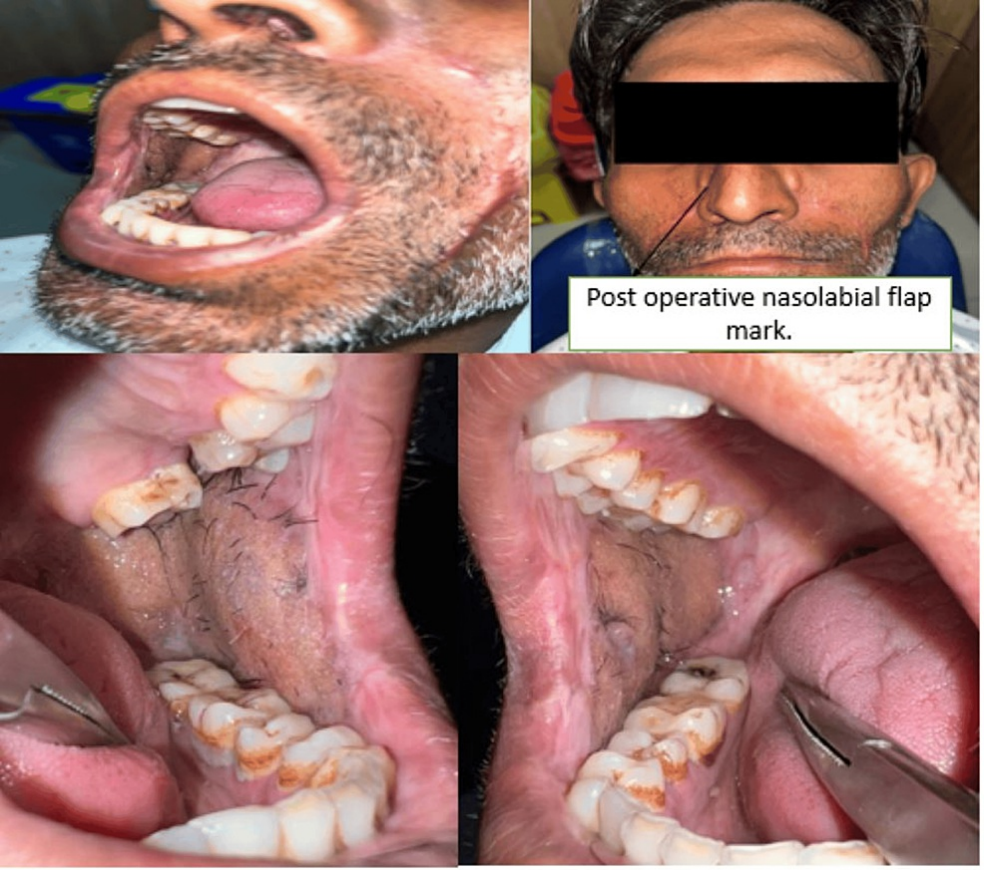
Post-operative images of extraoral scar and intraoral repair of the patient

**Figure 5:**
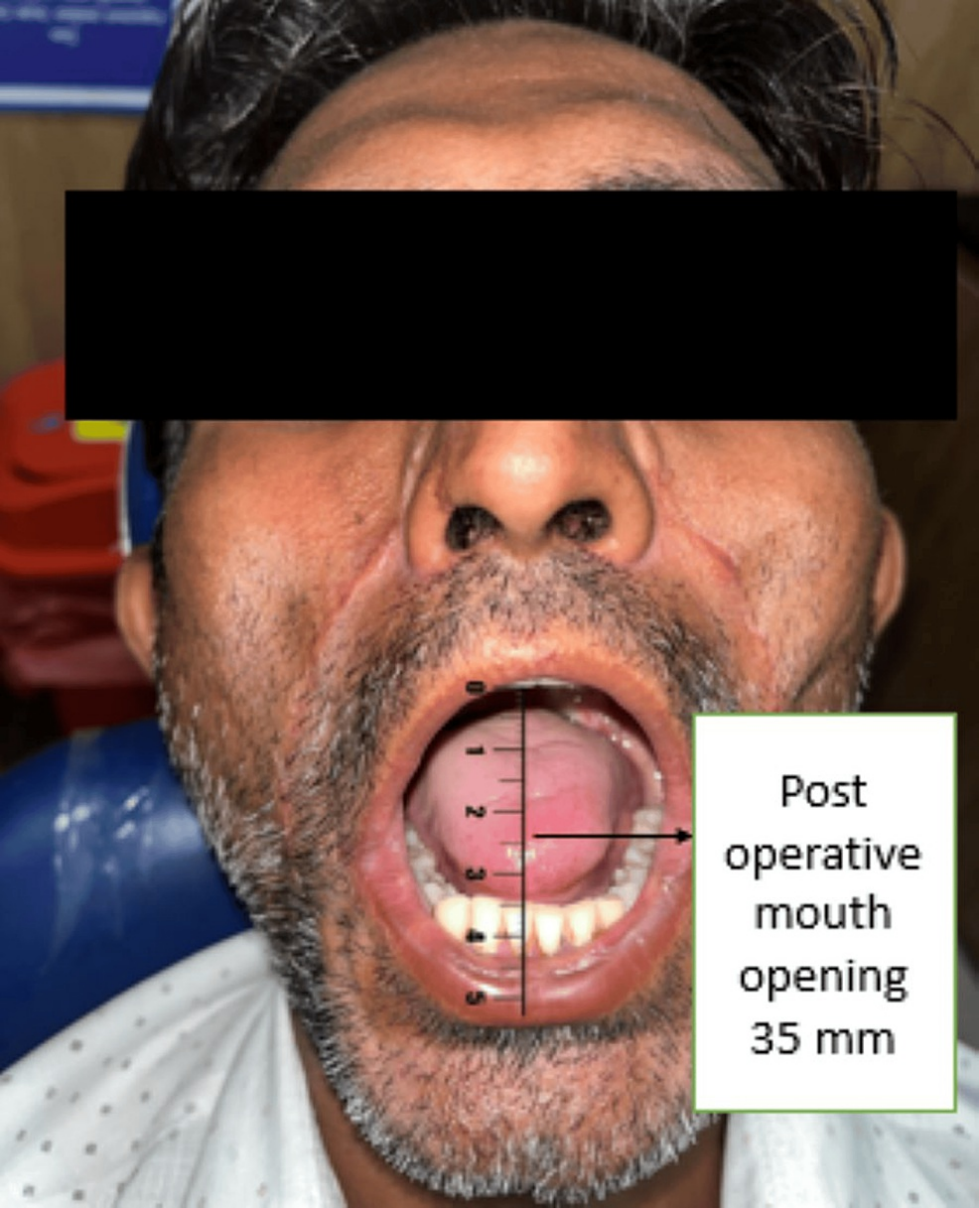
Post-operative mouth opening after complete treatment and physiotherapy

**Figure 6:**
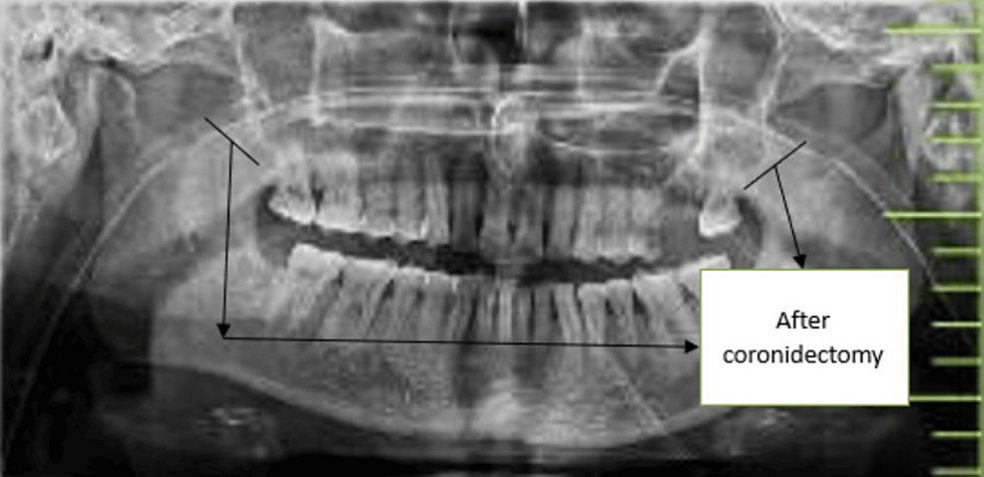
Post-operative orthopantomography showing reduction in coronoidectomy

The patient was reversed out of anaesthesia after the surgery, extubated, and then sent to the Surgical Intensive Care Unit (SICU) for further observation (Figure 4). After initial healing of post-operative day (POD) three, the patient has undergone a passive physiotherapy for an increase in mouth opening, which was achieved further actively after post-operative day seven. Figure 5 shows mouth opening post-operatively, and Figure 6 shows post-operative orthopantomography.

## Discussion

The involvement of areca nut as a key etiological component in oral submucous fibrosis has gained more attention in recent years; among those diagnosed with OSMF, the reported frequencies of areca nut chewing habit range from 84% to 100% [9,10]. Compared to home-prepared typical betel quid that contains lesser amounts of areca nut, commercially freeze-dried items like pan masala, Guthka, and mawa (areca and lime) tend to promote oral submucous fibrosis more quickly due to their high concentration of areca nut per chew. It seems sense to speculate that either decreased collagen breakdown or enhanced collagen synthesis might be contributing factors to the illness's onset. OSMF patients commonly seek medical assistance primarily due to the difficulty in mouth opening caused by oral mucosal rigidity. Khanna and Andrade in 1995 developed classification for oral submucous fibrosis depending on both histological features and clinical presentation of symptoms which is shown in Table 2 [7]. As a result, historical approaches to OSMF treatment have predominantly focused on improving mouth opening, reversing the altered oral mucosa, and improving the patient’s overall quality of life through either physiotherapy, medical interventions, or surgical procedures. Clinical management options for OSMF patients are typically categorized into nonsurgical or surgical modalities.

**Table 2:** Classification of oral submucous fibrosis given by Khanna and Andarde in 1995

Grades of oral submucous fibrosis	Mouth opening present
Grade 1 (very early cases)	No associated mouth limitation
Grade 2 (early cases)	Interincisal mouth opening is 26-35 mm
Grade 3 (moderately advanced cases)	Interincisal mouth opening 15-25 mm
Grade 4A (advanced cases)	Interincisal mouth opening less than 15 mm
Grade 4B (advanced cases)	Malignant transformation is seen in this stage

Conservative measures are preferred for patients whose mouth opening is more than 25 mm. Physical therapy and medical intervention are usually included in these. In physical therapy, splints or mouth opening devices may be used. In addition to routine exercise, according to some studies, hyperbaric oxygen therapy (HBOT) has the potential to induce fibroblast apoptosis and suppress fibroblast function by reducing the secretion of proinflammatory cytokines like interlukin-1 [11,12].

OSMF is treated using a range of medications classified as vascularity and inflammatory modulators, in addition to dietary supplements and adjuvant therapies. Steroids, interferon gamma, placental extracts, colchicine, anti-inflammatory and anti-fibrotic medicines, and intralesional injections and immunized milk are among the main players in the field of inflammation regulation, since they target inflammatory pathways to reduce the severity of fibrotic processes. Medication that addresses vascularity or ischemia relief, such as nylidrin, pentoxifylline, and buflomedil hydrochloride, aims to reduce tissue hypoxia and enhance blood flow, which slows the advancement of fibrosis. The overall effectiveness of therapy is enhanced by nutritional supplements that include beta-carotene, lycopene, vitamins, and minerals. These supplements assist antioxidant defence systems and tissue healing. Further, in more severe cases of oral submucous fibrosis, supplementary medications such collagenase, hyaluronidase, and chymotrypsin may be used to improve tissue remodelling and aid in functional restoration [13-15].

Surgical intervention is generally indicated when the mouth opening is less than 25 mm. These may include fibrotomy, coronoidectomy, and/or myotomy. To prevent re-fibrosis and promote healing without excessive scarring, various flaps and graft techniques are incorporated in the gap created in between. Different grafts that can be used are split skin grafts, collagen membranes, artificial dermis, and human amniotic membrane [16]. Flaps can also be used which are further classified as intraoral or extraoral flaps. Intraoral flaps include palatal island flap, buccal pad of fat, and tongue flaps, and extraoral flaps include nasolabial flap, platysma mucocutaneous muscle flap, submental artery based flap and temporalis myofascial flap, and free flap like radial forearm free flap [17].

There are some associated pitfalls of above said reconstructive modalities listed in the literature. There is high incidence of surgical contractual recurrence rate after split-thickness skin graft and uncertainty towards uptake of full-thickness skin graft [18]. According to Khanna et al., the use of island palatal flap is very constrained because of donor site fibrosis, limited tissue availability, restricted flap mobility, and requirement of extraction of teeth for accommodation of reach of flap with tension free manner [19]. Bilateral tongue flaps pose significant challenges, such as severe dysphagia, speech difficulties, and the risk of postoperative aspiration. Additionally, tongue flap and buccal pad of fat offer limited donor tissue and are prone to stability issues and dehiscence due to uncontrolled tongue movements. Furthermore, tongue involvement, reported at 38%, often prohibits its utilization for reconstruction purposes [18,20]. Among these, a nasolabial flap has been highly recommended and widely used because of its improved success rate and minimal technique sensitiveness [18].

In our institute, we frequently use it in practice. Even though it leaves an extraoral scar, it is usually less apparent, especially when the patient smiles, since it blends in with the natural nasolabial creases. In the present case report, we have treated a grade IV OSMF case with surgical intervention with bilateral fibrotomy, bilateral coronoidectomy, and reconstruction with bilateral bi-winged nasolabial flap. Intraoperative 40-mm interincisal mouth opening was achieved.

In addition to its pivotal role in achieving sufficient mouth opening and mitigating trismus recurrence post-surgery, rigorous mouth opening exercises serve multifaceted purposes within the context of oral rehabilitation [21,17]. Oral physiotherapy typically commences approximately three days post-surgery, strategically aligned with the initial phases of wound healing. Passive mouth opening (MO) exercise, employing wooden ice-cream sticks positioned between the molar teeth, patients incrementally expand their mouth opening capacity. Optimal therapeutic efficacy is achieved through a regimen characterized by sustained sessions lasting a minimum of three minutes, repeated three times daily, over a duration of at least three months augmented with active MO exercise with Heister’s jaw opening apparatus [17]. Adherence to this prescribed protocol is critical not only for consolidating functional gains but also for effecting behavioural change, particularly in discontinuing the consumption of areca nut, a habit integral to preventing fibrosis recurrence. Additionally, vigilant surveillance and regular follow-up for signs indicative of malignant transformation remains indispensable, underscoring the comprehensive nature of post-operative care in oral submucous fibrosis management.

## Conclusions

This case report demonstrates the advance features and prompt management of grade IV oral submucous fibrosis, a condition manifested by significant fibrosis and functional impairment of the oral mucosa. Advanced symptoms in the patient included significant trismus, mucosal stiffness, and burning sensation leading to difficulty in mastication and swallowing, highlighting the progressive nature of the condition. Despite the challenges posed by the advanced stage of OSMF, multimodal therapy involving dental, medical, and surgical procedures was carried out to alleviate symptoms, reversal of oral health, and decrease the chance of malignant transformation. However, the prognosis for patients with grade IV OSMF remains uncertain because of varied outcomes with respect to treatment options and due to poor patient compliance post-operatively. This case highlights how important it is to recognize OSMF signs early and take appropriate steps to limit the progression of the illness and lower the morbidity. It also highlights the need for continued study into the aetiology, risk factors, and the best treatment modalities for OSMF to enhance patient outcomes and overall quality of life. Even with advanced OSMF's challenges, a comprehensive and multidisciplinary strategy is still necessary to treat this debilitating condition.
